# A Potentially Ecosustainable Hazelnut/Carob-Based Spread

**DOI:** 10.1155/2024/4863035

**Published:** 2024-03-14

**Authors:** Laura Principato, Daniele Carullo, Alice Gruppi, Guillermo Duserm Garrido, Gianluca Giuberti, Milena Lambri, Giorgia Spigno, Andrea Bassani

**Affiliations:** DiSTAS-Department for Sustainable Food Process, Università Cattolica del Sacro Cuore, Piacenza 29121, Italy

## Abstract

Commercial cocoa and hazelnut-based sweet spreads typically present a poor nutribiochemical level due to their ingredients and recipes, while nowadays, there is the need of developing sustainable food products addressing both an improved nutritional and environmental profile. The aim of this work was then to develop an innovative hazelnut/carob-based spread with potential high sustainability and nutritional profile, including the exploitation of grape-processing residues (grape skin flour and grapeseed oil) and carob pulp as cocoa surrogate. Rheological (rotational/oscillatory), oxidative, and thermal features of the spread were assessed and compared with two commercial nut-cocoa-based products. Tribology was used to mimic and evaluate the spreads' behavior during oral consumption, and sensory profile (by quantitative descriptive analysis) was also assessed. All products exhibited a pseudoplastic behavior, with the elastic component prevailing over the viscous one. The innovative product showed the highest lubricity from both rheological and sensory analysis, thus well correlating to the obtained lowest viscosity and friction factor trends. Grapeseed oil provided a better nutritional profile, but the largest amount of unsaturated fatty acids promoted oxidation, despite the higher total phenolic content and antioxidant capacity coming from the use of carob and grape skin powders. The sensory perception investigation revealed a characteristic mouthfeel/flavor for the new spread identified having a more fluid consistency and a bitter/sour taste, together with a greater stickiness and a poorer smoothness due to a higher fiber content and solid fat absence.

## 1. Introduction

Nowadays, the intensive demand in feeding a continuously growing population requires a transition towards more sustainable food system and food products, to restore and preserve ecosystems for the health of the planet for either present or future generations. This is at the basis of the One Health concept stating that the health of humans, animals, and the environment are inextricably linked [1]. In this scenario, the development of a new food product is a tough and long-winded process. From one side, the finished goods must fulfil several requirements to reach a successful result in terms of quality, safety, and technological aspect. From the other one, consumers are more and more interested in “sustainability” and “functionality.” Sustainability is a complex concept which includes effects of a system, such as the food system, on the environment, economy, and society. As described in the perspective paper [2], the food system needs will need a profound transformation towards sustainability involving critical innovations ranging from food production, land use, and emissions to improved diets and waste management.

Regarding functionality, reported in [3], functional food is a concept rather than a well-defined group of food products, the European consensus document on functional food proposed this definition: “A food can be regarded as functional if it is satisfactorily demonstrated to affect beneficially one or more target functions in the body, beyond adequate nutritional effects, in a way that is relevant to either improved stage of health and well-being and/or reduction of risk of disease. A functional food must remain food and it must demonstrate its effects in amounts that can normally be expected to be consumed in the diet: it is not a pill or a capsule, but part of the normal food pattern.” According to [3], agrifood markets tend simultaneously to formulate new functional foods but also to rediscover traditional flavors and tastes. Moreover, an increasing number of consumers pay more attention to where ingredients are coming from. In particular, the EU population prefers the use of raw materials available nationally and/or elsewhere in the community, as opposed to products imported from outside of the European Union. Another reason for the farm-to-fork approach is the intensive and unsustainable consumption of several crops from abroad. For example, cocoa is one of the most widely used ingredients in spread preparation. Unfortunately, the demand for cocoa has continuously been increasing for years, and a further increase could provoke damages for many developing countries because of their lack of facilities to answer to this massive agricultural demand [4].

To face problems with possible shortage of cocoa and, at the same, promote the return to tradition, the carob pod was recently selected as a cocoa replacer [5] even though already used in the past with this aim during war periods, when it was critical to get access to cocoa. Widely spread in the Mediterranean area, the usage of carob has been largely widened in the past decades, as also testified by the presence of several carob-based products on the market [5, 6]. Carob is counted among the so-called “super foods” for its outstanding nutritional profile given by the low-fat content, no presence of stimulants (such as caffeine, theobromine, and oxalic acid), high level of dietary fibers (20% on average by weight, w/w), and minerals that exert positive effects against several diseases [7]. This is one of the factors at the basis of the expected rise in the Global Carob Chocolate Market size by 2027 [8]. Furthermore, the conspicuous level of sucrose increases carob suitability for no-added-sugar preparations. Finally, the roasting process enhances both antioxidant activity and sensory properties that better resemble cocoa-like flavor [9].

Another hot topic in the food scenario is the valorisation of industrially derived food residues according to the need of waste reduction previously mentioned. An interesting case study is given by the production of grape-processing by-products. Grape is one of the mostly cultivated fruit crops in the world, with a reported consumption of 77.8 Mton in 2018, where about 57% (w/w) is processed into wine [10]. However, wine processing generates large amounts of solid residues, with grape skins (GS) and grape seeds (GSD) being the main fractions of the solid wastes (up to 60% w/w and 20-25% of the received grapes, on average) [11]. Interestingly, these residual fractions, if properly exploited, could find large applications in the food, feed, pharmaceutical, and cosmetic industrial sectors [12, 13]. Specifically, GS contain considerable amounts of dietary fibers (43% w/w) and phenolic compounds with outstanding antioxidant activity [14]. Conversely, GSD are mainly used to produce oil that is renowned for its high nutritional profile due to the great amount of unsaturated fatty acids (14–20% of mono- (MUFAs) and 70% of polyunsaturated fatty acids (PUFAs)) and oil and polymeric proanthocyanins that prevent from several cardiovascular diseases [15–17].

The sustainability of cocoa or palm oil production has already been considered a hot topic at both industrial and academic levels. In addition, other trends such as sugar reduction, nutritional profile attention, and by-product valorisation have been currently taking place within the industrial food system [9, 18].

This research work represents a breakthrough because it is simultaneously developed at different levels in terms of careful attention to ingredient selection, such as carob and grapeseed oil, to improve both sustainability and nutritional profile claims of an innovative sweet spreadable cream thanks to fat substitution, sugar reduction, and residue valorisation. Within this frame, [19] developed a spread substituting cocoa with carob but with palm oil as the lipid fraction, thus maintaining the typical formulation of commercial spreads. The latter products, in fact, are typically rich in refined ingredients, such as sugars and vegetable oil, with a consequent poor nutribiochemical level, suggesting a very limited consumption in the daily diet [20]. Furthermore, to the best of our knowledge, no studies that exploit the simultaneous use of grapeseed oil and grape skins in carob-based spread are currently available in the literature.

Therefore, the main purpose of this work was to develop an innovative carob/nut-based sweet spread with attention to environmental and social (nutritional) sustainability aspects. The environmental target was addressed by preferring locally (in our case in Italy) available ingredients (carob and hazelnuts) to imported ones (cocoa/cocoa powder and palm oil), and through the valorisation of grape-processing residues (skins and grapeseed oil). The nutritional target was addressed selecting and combining the ingredients to design a functional food with a high nutritional profile which displays “source of or high fiber” (Regulation (EC) No 1924/2006, lastly amended by Regulation (EU) No 1047/2012) and “high unsaturated fat” (Reg. 116/2010) nutritional claims.

Two different commercial products were chosen to compare the newly developed product. The commercial products were chosen following previous research by the authors [21] according to the following features: the first one was identified as the top market player (*S*_*B*_), while the second one was targeted as a very high hazelnut content spread containing a 45% of hazelnut compared to the 13% of the market leader and many other cocoa/nut-based spreads on the market (*S*_*C*_). The macronutrient composition of our spread was evaluated and compared with the nutritional information of the selected market spreads. Additionally, for all the spreads, the total phenolic content and antioxidant capacity, rheological, tribological, and thermal profile, oxidative status and stability were assessed and compared. The sensory profile of all the spreads was evaluated through quantitative descriptive analysis (QDA) to get technical feedback from a trained panel.

## 2. Materials and Methods

### 2.1. Materials and Chemicals

Carob powder and grapeseed oil were purchased from LBG S.p.A. (Ragusa, Italy) and Benvolio S.p.A. (Treviso, Italy), respectively, while NoviLab Food S.r.l (Pozzolo Formigaro, Italy) kindly supplied PGO hazelnuts of “Tonda Gentile” variety, cultivated in Piedmont region in 2017 and industrial liquid, not GMO soy lecithin. Wine processing by-products, mainly composed of skins, were gently provided by a winery located in Tuscany (Italy). Grape pomace was obtained upon industrial pressing of red grapes belonging to both Merlot and Sangiovese varieties in the 2017 vintage. Grape pomace was treated in vibrating screens to separate skins from seeds. Afterwards, the final product (grape skins) was dried at 55°C (to limit antioxidant degradation) to reach a water content lower than 8% and milled to obtain a powder (mean dimension < 0.25 mm), which was packed in airtight bags and kept in a dark place until its use. Sugar beet white sugar (Eridania, Italy) and soy lecithin (Cereal, Spain) were purchased from a local supermarket, as well as two commercial cocoa/hazelnut spreads (SB and SC). The latter products, identified as control samples, were selected being different in terms of fat source (hazelnut/palm oil and hazelnut/cocoa butter, respectively), to be compared with the new formulation characterized, instead, by the absence of saturated fat content (Table S1). They were purchased with 10 months of residual shelf-life out of the overall 12 months of best by date and analyzed by one month from the purchase.

Petroleum ether, methanol, and chloroform were purchased from Titolchimica S.p.A. (Pontecchio Polesine, Italy); sulfuric acid, boric acid, hydrogen chloride, calcium carbonate, hexane, acetic acid, potassium iodide, isooctane, sodium carbonate, iron (III) chloride hexahydrate, sodium acetate, and iron (II) sulfate were obtained from Carlo Erba Reagents S.r.l. (Cornaredo, Italy); lead acetate, boron trifluoride, sodium thiosulfate, p-anisidine, Folin-Ciocalteu reagent, gallic acid, TPTZ (2,4,6-tripyridyl-s-triazine), and Mixed Indicator 5 were purchased from Merck KGaA (Darmstadt, Germany); ABTS (2,2′-azino-bis(3-ethylbenzothiazoline-6-sulfonic acid) diammonium salt) and Trolox® (6-hydroxy-2,5,7,8-tetramethylchromane-2-carboxylic acid, Sigma-Aldrich) were purchased from Sigma-Aldrich (Milan, Italy); Kjeldahl copper catalyst tablets were obtained from VWR International (Radnor, USA).

### 2.2. Spread Preparation and Experimental Protocol

The experimental protocol is shown in Figure S1 of the Supplementary Material. For the preparation of the *S*_*A*_ spread, carob flour (38.5%), hazelnut paste (34%), grapeseed oil (17%), sugar (8%), grape skin powder (2%), and soy lecithin (0.5%) were weighed at fixed proportions and refined. The refining step was executed to reduce the particle size and consequently promote spread smoothness and mouthfeel. The refining time and temperature were set at 50 min and 30°C, and the process was carried out in a pilot plant (NoviLab Food S.r.l.). The final *S*_*A*_ spread was packed in 200 g PET (polyethylene terephthalate) containers, which were subsequently stored at 20°C for further analyses.

The developed spread *S*_*A*_ was characterized for the composition in macronutrients and fatty acid composition to be compared with the nutritional information of the commercial products. The experimental and commercial spreads were then characterized for water activity, oxidative status of the extracted lipid fraction (based on evaluation of the content of number of peroxides, p-anisidine, conjugated dienes, and trienes), oxidation stability (based on the Oxitest system for both the extracted lipid fractions and the whole spreads), total phenolic content (based on Folin-Ciocalteu assay) and antioxidant capacity (based on the ferric reducing antioxidant power (FRAP) and ABTS assays) of the defatted fractions, thermal profile of both the extracted lipid fractions and the whole spreads (based on differential scanning calorimetry (DSC)), rheological properties (based on rotational and oscillatory rheology and on tribology), and sensory profile (based on Quantitative Descriptive Analysis (QDA)).

### 2.3. Composition in Macronutrients

Moisture, total protein, total fat, total dietary fiber, and sugar content were experimentally evaluated only for the developed spread.

Moisture content was evaluated as weight loss at 105°C ± 1°C for 24 h (AOAC's official method 931.04). The dried residue was placed in a preheated (550°C ± 10°C) muffle furnace to be subjected to the ash content determination.

Total fat content was evaluated by solid-liquid extraction of a 5 g sample with petroleum ether for 6 hours at 100°C (AOAC Method 945.16).

Total protein content was assessed based on total nitrogen content with the Kjeldahl method applying 6.25 as conversion coefficient [22].

Total dietary fiber and its fractions (soluble and insoluble ones) were determined using a K-TDFR-200A Kit (Megazyme, Wicklow, Ireland) based on the AOAC method 991.43 as reported by [23].

The sugar content was assessed by mixing 5 g of sample with 100 mL of distilled water, 1 mL of lead acetate solution, and 1 g of calcium carbonate for 15 min in a 200 mL flask and then filtered on carbon (this step is required to remove color and phenolic compounds that can interfere with the analysis). Afterward, the solution was made up to the mark (200 mL), and 50 mL was transferred in a 100 mL flask containing 5 mL of 2 N HCl and transferred in a water bath at 70°C for 15 min to carry out sucrose inversion. The reducing sugar content was determined according to the Fehling assay, adopting a Crison compact titrator (Crison Instruments SA, Alella, Spain). The analysis was carried out before and after acid inversion to estimate total sugars as the sum of sucrose and free-reducing sugars. Carbohydrate content was estimated by difference as reported in

(1) $$\begin{array}{c} \text{Carbohydrates}\%=100-\left(\text{proteins}+\text{fibers}+\text{lipids}+\text{ash}+\text{moisture}\right). \end{array}$$

### 2.4. Water Activity

For the analysis of water activity, approximately 5 g of each spread was weighed and placed in plastic containers to be analyzed with a water activity (aw) detector (Hygropalm, Rotronic, Bassersdorf, Switzerland).

### 2.5. Separation of Lipid and Defatted Fractions

A cold spread defatting procedure was applied to avoid oxidation of either the lipid or not-lipid components before subsequent analyses. Spreads were extracted using hexane as a solvent while maintaining a constant spread/solvent ratio of 1 : 5 (g/mL). Mixtures were homogenized for 3 min on a Vortex (Techno-Lab s.r.l, Brescia, Italy), placed on an orbital shaker (Ski 4, Argo Lab L.T.D, London) at 20°C for 1 h, and subsequently centrifuged (SL 16R, Thermo Fisher Scientific, Waltham, USA) at 4°C and 9200 g for 15 min. Finally, a rotating evaporator (BÜCHI Labortechnik GmbH, Essen, Germany) was used for hexane removal from the organic top phase at ambient temperature and under vacuum. The defatted fractions were let dry under nitrogen flow for one night. Three repetitions for each spread were carried out to recover the whole lipid fraction and the defatted fraction.

### 2.6. Total Phenolic Content and Antioxidant Capacity

The defatted fraction was subjected to phenolic extraction as reported by [24]. Briefly, 2 g of each defatted fraction was mixed with 50 mL of 80% methanol solution (v/v) using an orbital shaker (Shaker Incubator SKI 4, Argo Lab, Carpi, IT) at 180 rpm and 50°C for 2 h. Samples were then filtered on filter papers, with the liquid extract being collected in 50 mL flasks and made up to mark with methanol solution, prior to being stored for analysis.

The total phenolic content (TPC) of methanolic extracts was determined using the Folin-Ciocalteu assay protocol reported by [25], while the antioxidant capacity of the samples was evaluated through both FRAP and ABTS assays, following the methodology reported by [26]. The TPC was expressed as mg of gallic acid equivalents (GAE-Folin) per g of spread. Conversely, total antioxidant capacity results, obtained from the FRAP assay, were reported as mmol of reducing ferric(II) per moles of gallic acid equivalents (mol_Fe(II)_/mol_GAE_, FRAP), as well as micromoles of reducing ferric(II) per g of spread (*μ*mol_Fe(II)_/g_sample_, FRAP′). Similarly, ABTS outcomes were expressed as Trolox equivalent antioxidant capacity (TEAC, mol_Trolox_/mol_GAE_) or micromoles of Trolox per g of sample (*μ*mol_Trolox_/g_sample_ TEAC′) through a calibration curve obtained with Trolox between 0.1 and 2.2 mM.

### 2.7. Fatty Acid Composition and Oxidative Status of Lipid Fractions

The fatty acid composition of the developed spread lipid fraction was analyzed by gas chromatography after esterification with 14% (w/v) BF_3_ in methanol according to the method reported by [27].

The oxidative status of the lipid fraction of experimental and commercial spreads was evaluated based on the peroxides value (PV), conjugated dienes and trienes, and p-anisidine value. The PV was used to determine the presence of primary oxidation products while conjugated dienes and trienes measurement was carried out to detect secondary and tertiary oxidation products, respectively. PV, conjugated dienes (K_232_), and trienes (K_270_) were determined according to AOCS Official Methods Ca 5a-40, Cd 8-53, and Ch 5-9 1, respectively, as previously reported by [28].

The p-anisidine value (K_350_) determines aldehydes and ketones (principally 2-alkenals and 2,4-dienals) in oils and fat, according to the AOCS Official Method Cd 18-90 method as previously reported by [29].

### 2.8. Oxidative Stability

Accelerated oxidation tests were carried out on both whole spreads and lipid fractions using the Oxidation Test Reactor, Oxitest (VELP Scientifica, Milan, Italy), under high-oxygen level and temperature conditions. The Oxitest method has been recognized as an AOCS International Standard Procedure (AOCS Cd 12c-16) as mentioned by [30]. The instrument measures the pressure changes due to oxygen absorption by the food matrix. For the analysis, 45 g of sample was placed into the Oxitest chamber, working at 100°C and 6 bar of pressure. The Oxitest allows estimating the induction period (IP), defined as the time at which fat oxidation starts occurring. IP is denoted by a drop in O_2_ pressure due to oxygen consumption.

### 2.9. Thermal Profile

A micro-DSC calorimeter (Setaram, Caluire, France) was used to reveal the thermal behavior of either spreads or their lipid fraction during cooling/heating cycles. For the sake of analysis, 0.6 g of each sample was weighed and placed in Hastelloy capsules. An empty capsule of equal weight was used as a reference. The scanning temperature was raised from 30°C to 180°C at a rate of 0.8°C/min. As far as the sugar-melting phenomenon is concerned, the onset (*T*_onset_) and the offset (*T*_offset_) temperatures are defined as the intersection of the tangents of the peak with the extrapolated baseline, and the peak melting temperature (*T*_peak_) is defined as the temperature at the maximum/minimum of the thermal event. The analysis also allowed evaluating the induction temperature (IT) of lipid oxidation in nonisothermal conditions.

### 2.10. Rheological Measurements

Rotational and oscillatory measurements were carried out using a controlled-stress MCR-302 rheometer (Anton Paar, Graz, Austria) provided with a rough plates' geometry. The plates were 25 mm in diameter, and a 1 mm gap was selected to perform the tests. The rheological behavior of all spreads was analyzed in either static (rotational) or dynamic (oscillatory) conditions. The applied methods were the same as reported in [23] with slight modifications.

#### 2.10.1. Rotational Rheology

Rotational measurements were performed at 10°C, 30°C, and 60°C to assess the dependency of spread viscosity (*η*) on temperature. The shear rate ($\dot{\gamma }$) was set between 0.01 s^−1^ and 100 s^−1^, with the flow curves (*η* vs $\dot{\gamma }$) being analyzed using the power law model to describe the rheological behavior of the investigated samples.

#### 2.10.2. Oscillatory Rheology

The linear viscoelastic region (LVR) for the tested spreads was obtained through the amplitude strain sweep test in which the frequency value was kept constant (1 Hz) while the amplitude was varied within the range of 0.01-100%. For frequency sweep tests, performed at a constant shear strain of 0.02%, values from 100 to 0.1 Hz were considered. The viscoelastic parameters (storage modulus, *G*′, loss modulus *G*^″^, and loss tangent, tan*δ*) were plotted versus the frequency. Measurements were carried out at 40°C (close to the oral cavity temperature of 36.5°C used for the tribological measurements).

#### 2.10.3. Tribological Measurements

As reported in [23], a tribocell equipped with a stainless-steel ball-on-three-PDMS-plate geometry was used to evaluate the friction coefficient of the spread at 36.5°C (the average oral cavity temperature). Before the analysis, each sample was maintained and equilibrated at 36.5°C for 1 hour to achieve a homogenous temperature in the spread. The measurement was carried out for 20 min, applying a constant force of 5 N and under variation of the sliding velocity from 0.01 to 1000 mm/s. The results were obtained as variation of the friction coefficient as a function of the sliding speed (Stribeck curve).

### 2.11. Sensory Profile (Quantitative Descriptive Analysis)

The sensory profile of the spreads was determined using QDA [31] with a trained panel (*n* = 30, equal number of males and females with declared routinely consumption of this kind of products). Based on a previous study [32], twenty sensory attributes were selected and inserted into an evaluative papery scorecard: brown color, shininess, spreadability, homogeneity, hazelnut taste, cocoa taste, roasted taste, fatness, adhesiveness, solubility, viscosity, lubricity, sweetness, bitterness, sourness, hazelnut flavor, cocoa flavor, roasted flavor, persistency, and complexity. Panelists were instructed to taste all the samples with the provided teaspoons. Before analysis, each panelist completed 11 h of training according to [33] procedure, with slight modifications. The intensity of each attribute was scored on a nine-point horizontally oriented scale anchored as “not perceived at all” and “extremely intense” at the left and right ends, respectively. Samples were served in 30 mL portions inside white cups labeled with random three-digit blinding codes. Mineral water and white napkins were provided to avoid carry-overs among samples which were monadically served to panelists every 10 min. The order of presentation was randomized across the panelists. All responses were recorded and represented in a traditional QDA spider chart.

### 2.12. Statistical Analysis

All analyses were made at least in triplicate, and data are reported as mean values and standard deviations (SD). The influence of spread type on rheological/tribological characteristics was evaluated using a one-way analysis of variance (ANOVA) followed by Tukey's post hoc test for mean discrimination (*p* ≤ 0.05). The statistical analysis was executed via the IBM SPSS Statistics 21 software (SPSS Inc., Chicago, IL, USA).

## 3. Results and Discussion

### 3.1. Composition in Macronutrients

The ingredient list and the nutritional information for both innovative (*S*_*A*_) and commercial (*S*_*B*_ and *S*_*C*_) spreads are reported in Table S1. The formulation of *S*_*A*_ was the result of preliminary tests (not reported here) in which different percentages of the selected ingredients were combined considering the list of ingredients of commercial products, the target nutritional claims “source of or high fiber” and “high unsaturated,” and testing different carob flours of different roasting levels. A specific mix of carob flour with a certain roasting level and not roasted carob flour was selected to get a color and aroma very close to extra dark chocolate spread (from the roasted flour) and some sweetness (from the raw flour). It was not possible to increase the amount of grape skin powder above 2%, nor to reduce the sugar addition below 8% due to astringency/bitterness perceptions. *S*_*A*_ shows a carbohydrate and sugar content 2.26-fold and 1.69-fold lower than *S*_*B*_ and *S*_*C*_, respectively. The lower sugar addition for our produced spread is also visible from the ingredient list order in which, for both *S*_*B*_ and *S*_*C*_, sugar is reported as first and second one, respectively, while for *S*_*A*_, sugar is at the very end of the ingredient list. An opposite trend is highlighted for lipid content for which *S*_*A*_ shows the highest one due to the use of hazelnut paste (34%) and grapeseed oil as main ingredients, with hazelnuts having a lipid content ranging from 40% to 60% (w/w), depending on the variety [34]. As far as *S*_*B*_ is regarded, even if palm oil is placed as the second ingredient, the hazelnut amount is very low (13%), and, if the ratio between sugar and lipid content is considered, it is clear how sugar is the major ingredient. On the other hand, *S*_*C*_ contains the highest amount of hazelnut (45%), and cocoa is mentioned at the end of the ingredient list indicating a low dosage.

It is also worth noting that, despite the fat content of *S*_*A*_ being slightly higher than that of *S*_*B*_ and *S*_*C*_, the fatty acid composition analysis reported in Table S2 revealed the presence of mainly unsaturated fatty acids (overall 88.64 g/100 g total fatty acids), of which 57% monounsaturated and 43% polyunsaturated fatty acids, and a reasonable amount (37.91 g/100 g total fatty acids) of the *ω*-6 linoleic acid, as the main component of the lipid fraction. Therefore, the fatty acid profile of *S*_*A*_ would guarantee the “high unsaturated fat” nutritional claim (EU Reg. 116/2010) which requires that “at least 70% of the fatty acids present in the product derive from unsaturated fat under the condition that unsaturated fat provides more than 20% of energy of the product.”

Another valuable result for *S*_*A*_ is its highest amount in dietary fibers due to the use of carob flour (Table S1). This is because carob pulp, used for the flour production, is rich in insoluble dietary fiber. It is also reported to be high in polyphenols that display high antioxidant capacity, accompanied by a series of potential benefits on health, such as lowering serum cholesterol and serum triglycerides, as well as assisting the postprandial lipid metabolism [6, 19]. According to EU Reg. 1924/2006, the fiber level assessed in *S*_*A*_ formulation would be enough for the nutritional claim “high fiber” (“the product contains at least 6 g of fiber per 100 g or at least 3 g of fiber per 100 kcal), thus confirming the good nutritional profile of our newly produced spread.

### 3.2. Total Phenolic Content and Antioxidant Capacity

Data related to TPC are reported in Table 1. *S*_*A*_ revealed the highest phenolic content, approximately 4-fold and 5-fold higher than *S*_*B*_ and *S*_*C*_, respectively. Such results can be reasonably linked to the *S*_*A*_ formulation with carob pulp, hazelnut, grapeseed flour, and grapeseed oil, being all sources of antioxidant compounds. Conversely, *S*_*B*_ and *S*_*C*_ derive their phenolic content from cocoa or hazelnut paste only. In this regard, *S*_*B*_ contains a very small amount of cocoa and hazelnut ingredients, justifying the lowest observed value. On the other hand, *S*_*C*_ displayed a high hazelnut content (45%, w/w) comparable to *S*_*A*_, but TPC was lower revealing that this ingredient plays a minor role than carob- or grape-based ingredients, in providing phenolic compounds.

The found TPC values are in line with those reported by [35] with a 1.95 mg_GAE_/g TPC for a spread with 8% cocoa powder that could be increased up to 4.4 mg_GAE_/g TPC with the incorporation of 15% hazelnut cake.

Table 1 reports also the antioxidant capacity results. No statistical differences in terms of specific antioxidant capacity (TEAC_ABTS_, FRAP) were detected, hence confirming that polyphenols strongly contribute to the antioxidant power of considered samples. On the other hand, when the antioxidant capacity is expressed on a sample mass basis (TEAC′_ABTS_, FRAP′), *S*_*A*_ exhibited the highest activity level (*p* < 0.05) with respect to both *S*_*B*_ and *S*_*C*_ in agreement with the total phenolic content (it must be said that the Folin assay is, anyway, an analytical method based on the reducing capacity of the sample).

### 3.3. Oxidative Status and Oxidative Stability

The three spreads showed, as expected, low and statistically not different water activity values, being 0.525 ± 0.016, 0.533 ± 0.025, and 0.526 ± 0.006 for *S*_*A*_, *S*_*B*_, and *S*_*C*_, respectively. The products are, then, stable from a microbiological point of view, and the main degradation will occur due to fat oxidation. The oxidative quality of the lipid fraction of the spreads was assessed measuring the conjugated dienes and triene content, the peroxides value, and the p-anisidine content (Table 2). Our new spread, *S*_*A*_, showed the highest diene and triene content, as it was expected due to the higher content in lipids and in unsaturated fats. Whatever the analyzed spread, no detectable result was recorded for peroxides showing that the manufacturing process did not alter the grapeseed oil for *S*_*A*_ and confirming the freshness of the two commercial spreads (as specified in Materials and Methods, they were purchased with 10 months of residual shelf-life out of the overall 12 months of best by date). Furthermore, *S*_*B*_ did not show any recordable value for any of the measured parameters coherently with the use of palm oil in the recipe.

The oxidation stability tests performed with the Oxitest reactor were carried out to compare the three spreads in terms of oxidation stability, even though in the literature, there is no available demonstration of the exact correspondence between the test conditions (pure oxygen environment and high temperature) and the ideal suggested storage conditions (in closed barrier packaging and 18-22°C). The analysis should provide a result of the oxidation induction period (IP) which is related to the inflection point of curves. However, when measurements were performed directly on the spread, no inflection could be detected, but rather a continuously decreasing oxygen pressure curve (data not shown). For this reason, we carried out the analysis on the extract lipid fractions, even though this removes the protective effect of the matrix against oxidation. As visible from Figure S2, the lipid fraction of *S*_*A*_, *S*_*B*_, and *S*_*C*_ showed an induction period of 576 min, 1388 min, and 1269 h, respectively. The higher phenolic content and antioxidant capacity of the *S*_*A*_ formulation could not mitigate fat oxidation which occurred much earlier than in commercial spreads. *S*_*C*_ exhibited a slighter higher resistance to oxidation phenomena than *S*_*B*_ despite the lower saturated fat content, and this could be due to the very high content in hazelnuts (45%, w/w) which typically contain tocopherols with antioxidant activity [36].

### 3.4. Thermal Profile

From DSC thermograms, no fat melting phenomena were detected probably due to matrix effects that hide the signals (data not shown). Conversely, all samples exhibited an endothermic peak above 125°C, which could be associated with the melting phenomenon of sugars [37].

Table 3 presents DSC characteristic temperatures and enthalpies for sugar melting of both experimental and commercial spreads. Results showed that the peak temperature ranged from 125°C to 140°C for *S*_*A*_ and from 145 to 160°C for *S*_*B*_ and *S*_*C*_. Such significantly different onset and offset points may be related to the specific typology of sugars involved within product formulation. In fact, while sucrose is one important ingredient in *S*_*B*_ and *S*_*C*_, *S*_*A*_ mainly contains fructose and glucose deriving from carob pulp. As far as sugar-melting enthalpies are concerned, significant (*p* < 0.05) differences among mean values were also recorded. Specifically, *S*_*A*_ exhibited the smallest value (*p* < 0.05), which could be ascribed to the lowest sugar content followed by commercial spreads, in accordance with the nutritional composition of Table S1.

In agreement with the findings of [38], all samples were characterized by typical trends of lipid oxidation for which, at high temperatures (>150 > 150°C), the heat flow signal separates from the baseline (straight line) with a dramatic slope increase (data not shown). The temperature at which such a phenomenon occurs is known as the induction temperature (IT) and represents a stability indicator for lipid oxidation [39]. As clearly emerging from Table 3, *S*_*A*_ reported the lowest IT value, followed by *S*_*C*_ and *S*_*B*_, respectively. Again, induction temperature depends on total lipid fraction and solid fat content, as it was confirmed by the results from oscillatory rheology tests (see next section) and the lower oxidative stability of *S*_*A*_ from Oxitest analysis.

### 3.5. Rheological Measurements

#### 3.5.1. Rotational Rheology

**Figure 1:**
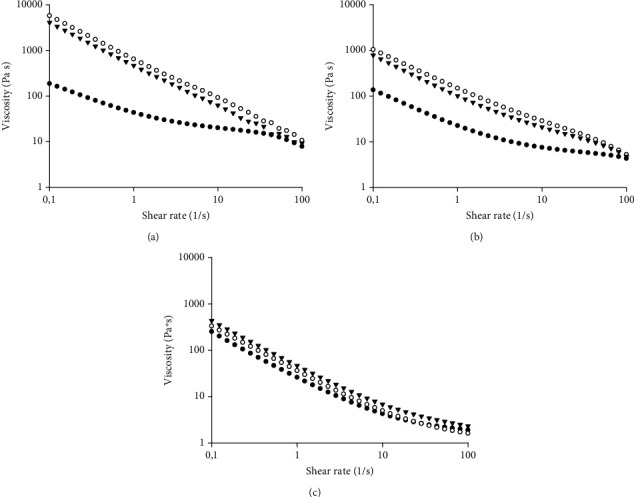
Curves of viscosity (*μ*, Pa·s) versus the shear rate ($\dot{\gamma }$, s^−1^) at (a) 10°C, (b) 30°C, and (c) 60°C obtained with plate-plate geometries for the tested spreads of Table S1: *S*_*A*_ (black circles), *S*_*B*_ (white circles), and *S*_*C*_ (black triangles).

Flow curves of tested spreads, measured at different temperatures, are reported in Figure 1. Regardless of the applied temperature, *S*_*A*_, *S*_*B*_, and *S*_*C*_ behaved as pseudoplastic fluids, with the apparent viscosity decreasing with increasing shear rate, in accordance with [21], in which the two commercial spreads were already analyzed and compared with additional commercial spreads. Evident discrepancies among spreads were highlighted at the lowest investigated temperature (Figure 1(a)), since at 10°C and low shear rates (0.1-1 s^−1^), *S*_*A*_ showed a viscosity value approximately a hundred times lower than that of *S*_*B*_ and *S*_*C*_. Instead, at high shear rate values (30–100 s^−1^), no appreciable difference among curves could be detected, being almost overlapped. At 30°C (Figure 1(b)), the gap present between curves characterizing our spread and the commercial ones dropped by ten times and, interestingly, almost disappeared at 60°C probably due to the full fat melting (Figure 1(c)). It is well known that viscosity is one of the main parameters affecting emulsion stability. In our case, *S*_*A*_ was extremely fluid with viscosity values ranging from one to two orders of magnitude lower than *S*_*B*_ and *S*_*C*_. Nevertheless, *S*_*A*_ seemed to maintain a completely unaltered structure upon temperature changes with respect to commercial spreads, for which temperature dependency appeared more pronounced.

To better investigate the influence of the temperature on rheological parameters, flow curves were fitted according to the Ostwald model, commonly referred to as the power law model in the linearized version (Equation (2)) as explained also in [21],

(2) $$\begin{array}{c} \log\eta =\log K+\left(n-1\right)\;\log\dot{\gamma ,} \end{array}$$

where *η* is the viscosity (Pa·s), $\dot{\gamma }$ is the shear rate (s^−1^), *K* is the consistency index (Pa·s^n^), and *n* is the dimensionless flow index.

According to the results deriving from the application of the power law regression (Table 4), whose goodness of fit was quite acceptable (*R*^2^ > 0.953), all spreads possessed *n* values lower than 1, thus confirming the previously observed shear thinning behavior (Figure 1), in agreement with [21] and some literature findings on nut or pistachio spreads [40–42]. However, independently on the temperature, *S*_*A*_ exhibited both *K* and *n* values significantly different (*p* < 0.05) as compared to *S*_*B*_ and *S*_*C*_. More specifically, *S*_*A*_ reported the highest values of *n*, thus indicating the most fluid tendency, and the lowest *K* values which, instead, confirmed a weaker structure. The observed discrepancies in terms of *K* and *n* parameters among tested spreads could be ascribed to the variability of fat composition and solid concentration in the products' formulation. According to the nutritional composition (Table S1), *S*_*A*_ contains the lowest amount of saturated fatty acids due to grapeseed oil presence. Indeed, vegetable oils behave like Newtonian fluids and remain in the liquid state at room temperature [43]. Grapeseed oil is rich in unsaturated fats, among which MUFAs and PUFAs (Table S2), as compared to palm oil and cocoa butter in which the saturated fat amount ranges from 50% to 65% (w/w) [44]. On the other hand, regarding the effect of solid concentration on the rheological behavior of food products, carbohydrates can bind water, and this interaction might affect solute conformation. A high amount of carbohydrate content, such as that reported for the investigated commercial spreads, dramatically reduces the free energy of the system and, consequently, increases product viscosity. Similar results were previously reported in the works of [45, 46], who observed a gradual increase of apparent viscosity when increasing °Brix content in mango puree and apple juice, respectively. Previous work [47] highlighted a strong effect of total soluble solid (TSS) concentration on the apparent viscosity of oat milk, thus showing a 145%, 504%, and 1388% increase in viscosity as the TSS increased from 5 to 10, 15, and 20 °Brix, respectively. Similarly, [48] found a strict direct dependency between total solid content and viscosity in their study on monitoring the total solid content of milk protein concentrate by using acoustic transmission technology.

From the results of Table 4, it is also noticeable that, for temperatures ranging from 10°C to 30°C, *S*_*B*_ showed the highest *K* values (*p* < 0.05) followed by *S*_*C*_ and *S*_*A*_, respectively. These results seem to follow the sugar composition reported in Table S1 and, hence, corroborate the aforementioned theory.

As done in [21], data of viscosity vs. temperature, evaluated at a shear rate of 50 s^−1^, was modeled by the Arrhenius law:

(3) $$\begin{array}{c} \eta =\eta_{\infty }e^{E_{a}/\text{RT}}, \end{array}$$

where *η* is the viscosity (Pa·s), *T* is the temperature (K), and *R*, *E*_*a*_, and *η*_∞_ are the gas constant (0.00831 kJ/(K·mol)), the Arrhenius activation energy (kJ/mol), and the preexponential factor of the Arrhenius equation for the liquid system (Pa·s), respectively. The logarithmic linearization of Equation (3) yielded an acceptable goodness of fit (Table 5).

Among all samples, *S*_*B*_ exhibited the highest value in terms of activation energy, followed by *S*_*C*_ and *S*_*A*_. Previous authors [49, 50] already found that thermal stability is related to the lipid fraction of samples. By decreasing fat content within the formulation, the food structure is more exposed to structural breakdown, which results in a greater *E*_*a*_ value. In our case, even if *S*_*B*_ contains the highest amount of saturated fats, the total lipid fraction is lower than that contained in *S*_*A*_ and *S*_*C*_, with the latter spreads being similar not only in terms of fat content but also for ingredient list (Table S1). The observed results might be related also to a more prominent fat melting phenomenon: consistency modification due to phase transition can justify the higher sensitivity to thermal changes expressed by *S*_*B*_ (Table 5).

#### 3.5.2. Oscillatory Rheology

**Figure 2:**
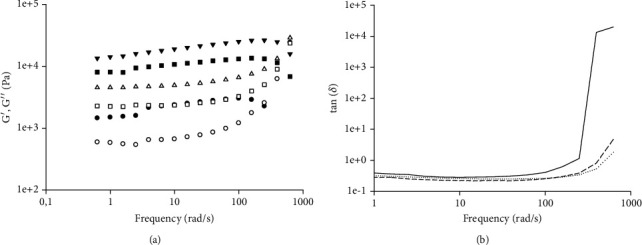
(a) Curves of storage modulus (*G*′) (black symbols) and loss modulus (*G*^″^) (white symbols) and (b) curves of tan (*δ*), obtained in the oscillatory regime as a function of the frequency, for the spreads of Table S1. For *G*′/*G*″ analysis: *S*_*A*_ (circles), *S*_*B*_ (triangles), and *S*_*C*_ (squares). For tan (*δ*) analysis: *S*_*A*_ (solid line), *S*_*B*_ (dotted line), and *S*_*C*_ (dashed line). Tests were carried out at 40°C.

With the purpose to gain insight into system stability, curves of storage modulus (*G*′) and loss modulus (*G*^″^) of all the tested samples, obtained from frequency sweep trials at 40°C, were obtained (Figure 2(a)). Regardless of the considered spread, results highlighted the predominant solid-like or highly structured material behavior over the viscous one (*G*′ > *G*^″^). Moreover, both viscoelastic moduli were dependent on frequency within the range of 1–200 rad/s and displayed typical weak gel characteristics [51, 52]. According to the findings of [53], the distance between *G*′ and *G*^″^ is strictly related to the strength of gels. Lower distances between moduli, as reported in the case of *S*_*A*_, underline a weaker structure. On the other hand, the greater space gap present in correspondence of *S*_*B*_ and *S*_*C*_ witnesses the likely presence of stronger colloidal forces. However, by increasing the frequency, materials become progressively more unstable with the fade of typical viscoelastic moduli evolution. When *G*^″^ value exceeds *G*′, curve superposition occurs forming the so-called cross-over point. Specifically, *S*_*A*_ exhibited the earliest cross-over (*w* = 200 rad/s) as compared to *S*_*B*_ and *S*_*C*_ for which, instead, such phenomenon shifted towards higher frequency values (*w* > 400 rad/s) (Figure 2(a)). The observed behavior could be attributed to the minor saturated fat content for *S*_*A*_. Another reason can be found in lower viscosity values since in a study on colloidal system stability [54], it was found that a lower value of viscosity was related to a higher tendency in phase separation and decreasing storage stability. Despite the inclusion of grapeseed oil within the formulation process having caused a reinforcement in the nutritional profile of *S*_*A*_, the consistent amount of unsaturated fatty acids might have contributed to producing a weaker structure due to their liquid state and, hence, the occurrence of an earlier cross-over. Conversely, the presence of palm oil in *S*_*B*_ and cocoa butter in *S*_*C*_ not only would improve structural consistency according to rotational rheology results (Figure 1), but it would also generate a delay in the mechanical breakdown.

The loss in colloidal strength is also corroborated by the tan*δ* trends, which are depicted in Figure 2(b). Among all samples, the *S*_*A*_ curve is placed the nearest to the threshold value (tan*δ* = 1), thus indicating that viscoelastic moduli possess comparable magnitude. As expected, at 200 rad/s of frequency, a dramatic slope increase occurred exceeding limit values and confirming structural weakening. In agreement with the previously commented results of Figure 2(a), *S*_*B*_ and *S*_*C*_ exceeded the threshold value at higher frequencies (Figure 2(b)).

#### 3.5.3. Tribological Measurements

**Figure 3:**
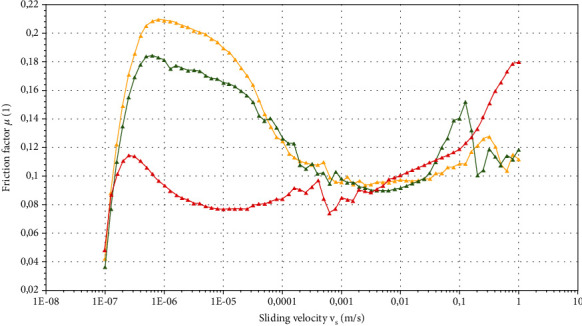
Stribeck curves obtained at 5 N load force, showing the effect of sliding velocity on the friction factor for the spreads of Table S1: *S*_*A*_ (red triangles), *S*_*B*_ (yellow triangles), and *S*_*C*_ (green triangles). Tests were carried out at oral cavity temperature (36.5°C).

Tribological results are summarized in Stribeck curves reported in Figure 3.

A Stribeck curve normally contains three different regions, namely, the boundary, the mixed, and the hydrodynamic regimes. In the former one, the friction is governed by the ability of the fluid to form a thin film between the surfaces, being independent of the sliding speed [55]. If the speed is increased, a hydrodynamic film is created, and the friction is significantly reduced (i.e., the Stribeck curve enters the mixed regime). Such reduction is governed by fluid lubricant properties and liquid-surface interactions that promote the entrainment [56]. For further speed increments, the hydrodynamic film is fully developed, and the surfaces are completely separated (i.e., occurrence of the hydrodynamic regime), with the friction linearly increasing with speed upon viscosity dependency [57]. Overall, the friction curves associated with the investigated spreads resembled a typical three-zone Stribeck plot (Figure 3). Peculiarly, in the boundary regime, each curve showed a plateau-like zone in which friction remains almost constant with sliding velocity. Friction magnitude for *S*_*B*_ and *S*_*C*_ was almost 2-fold greater than for *S*_*A*_. Moreover, the latter spread possessed a narrower boundary regime (7·10^−6^ m/s) than those related to *S*_*B*_ and *S*_*C*_, for which friction started declining at a greater sliding speed (2·10^−5^ m/s). We have already commented that *S*_*A*_ had the highest amount of unsaturated fats and the lowest value of viscosity. Both factors might affect material fluidity and positively promote lubricant capacity. Conversely, the more consistent and solid-like structure of *S*_*B*_ and *S*_*C*_ might contribute to a greater resistance to gap entrapment by causing the larger boundary regime tract (Figure 3). When sliding speed further increases, both mixed regime and hydrodynamic regime occur. Again, *S*_*A*_ developed the mixed regime earlier (8·10^−6^ m/s) than *S*_*B*_ and *S*_*C*_ (8·10^−4^ m/s). Additionally, in the mixed regime, the friction factor for commercial spreads dramatically dropped until approximately 50% of the initial value. Especially in the case of *S*_*B*_, a possible explanation for the detected trend can be linked to the highest sensitivity to thermal phenomena, as previously highlighted by the results from the Arrhenius model (Table 5). Heat generation during friction and temperature-induced melting phenomena of the solid fat content may promote the structural breakdown and a more fluid consistency. Nevertheless, similar friction can also be easily correlated with greater rupture forces. At higher sliding speed, mechanical stress promotes network disruption giving comparable fluidity. Finally, at greater velocity level (>10^−1^ m/s), another trade-off is highlighted, with *S*_*A*_ friction rising more severely as compared to *S*_*B*_ and *S*_*C*_ which, instead, are characterized by a smoother friction increase (Figure 3).

Moreover, from a macroscopical point of view, *S*_*A*_ presented a larger amount of material on the external wall of the tribological cell (data not shown) at the end of the test. This can be related to product phase separation occurring at very harsh conditions. Plus, product instability might have caused material slack due to structural breakdown and oil loss with consequent friction rise. Similar results were reported by [58] in their study on the emulsion stability of modified fish gelatin with gum Arabic and octenyl succinate anhydride gum Arabic, with and without glycosylation. Tribological data indicated that a glycosylated system could stabilize emulsions by reducing the shift towards higher friction values over time. The main phenomenon related to emulsion stabilization was found in protein-polysaccharides conjugates. Such macrocomplexes reduced the droplet-to-droplet interactions in emulsions, thus creating a pressure that supports separation of the interacting surfaces, reducing friction coefficient, and granting its lubrication properties [58]. These insights are also in line with oscillatory rheology for which *S*_*B*_ and *S*_*C*_ cross-over points are strongly delayed, suggesting greater product stability over time with respect to *S*_*A*_ (Figure 2(a)).

Another possible interaction phenomenon that might have affected the friction coefficient is adhesion. The relation between adhesion and tribology has been increasingly highlighted especially for polymers [59], but it can be also transferred to more complex systems. The simplest adhesion mechanisms are found considering the interlocking interaction between surface irregularities and product surface or looking at some specific molecular activities occurring within the contact area. Within this frame, [59] reported how cross-linking of polymer surfaces reduces both adhesion and friction.

### 3.6. Sensory Profile (QDA)

Development of a new product can be a very long process, starting from conception and proceeding to validation of proof of concept, process optimization and upscaling, shelf-life testing, and commercialization. Quality assessment and sensory evaluation are important along the whole process. In relation to sensory evaluation, both affective (subjective) tests and analytical (expert) tests can be used at every step of a new product development process. However, subjective rather than analytical sensory tests require different participants in terms of both skills and number [60], and then, the most appropriate sensory test should be selected depending on the main question you have to answer to. The aim of this work was to develop an innovative hazelnut/carob-based spread compared to commercial cocoa and hazelnut-based sweet spreads. In relation to sensory analysis, the objective was to determine the intensities of all product attributes, define its complete sensory profile, find correlations with recipe composition and other analytical measurements, and evaluate if and how the developed product differed from commercial products. All these aspects must be evaluated using descriptive tests, such as QDA, normally performed by 6 to 15 selected and trained panelists [60]. Selection of QDA is also linked to the fact that the new product was not yet in the final stage of acceptability and not yet ready to be submitted to untrained consumers for an acceptability test, which normally requires many not trained respondents (50-150 panelists considered adequate).

**Figure 4:**
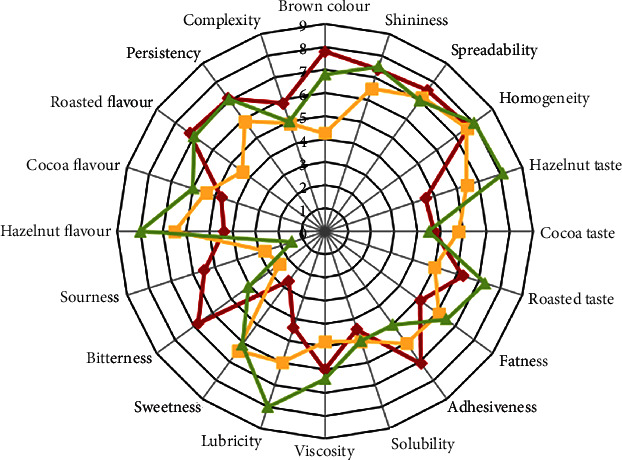
Spider web graph of flavor, taste, and texture attributes for the spreads of Table S1: *S*_*A*_ (red diamonds), *S*_*B*_ (yellow squares), and *S*_*C*_ (green triangles).

The spider chart reported in Figure 4 resumes all sensory attributes related to visual, olfactory, tactile-taste, and retroolfactory phases for the QDA of all the samples. From visual description analysis, *S*_*A*_ showed the highest score in brown color, while *S*_*C*_ reported similar results for homogeneity and shininess.

Interesting insights were found in the tactile-taste area. Within this frame, sweet perception was less pronounced for *S*_*A*_ compared to *S*_*C*_ and *S*_*B*_, in agreement with nutritional composition (Table S1), as well as DSC analysis results (Table 3). Nevertheless, the fattiness attribute was minimized for *S*_*A*_, even though the formulation contained the highest lipid content. However, if only solid fats are considered, it is possible to hypothesize that saturated ones dominate the perception mechanism (Table S1). Mouthfeel parameters highlight that *S*_*A*_ showed a characteristic bitter/sour taste and a richer and more complex profile after swallowing. No difference in terms of consistency, spreadability, and solubility was detected, even though rheological analysis reflected a significant (*p* < 0.05) variability among samples (Table 4). This may be explained by considering the interaction occurring between sample and saliva, which acts as a diluent and levels human perceptions during oral consumption. Moreover, panelists underlined the greatest stickiness and the lowest smoothness for *S*_*A*_. The latter result might be related to fiber content and no presence of solid fats in spread formulation.

The evident adhesion properties that were perceived by panelists can be linked with the increase in friction highlighted in the hydrodynamic regime of the Stribeck curve (Figure 3). In terms of olfactory profile, toasted flavor, cocoa, and nut aromas were evaluated. To this purpose, *S*_*C*_ possessed the highest score in terms of hazelnut flavor due to the consistent (45% w/w) hazelnut content (Table S1). Similarly, *S*_*A*_ contains 34% (w/w) of hazelnut, but roasted aromas (linked to the use of roasted carob flour) resulted to be preponderant on nut components, while the cocoa flavor was estimated to be dominant for *S*_*B*_.

## 4. Conclusions

This study explored the possibility to develop a sweet carob/hazelnut spread with a potentially higher environmental sustainability and better nutribiochemical level than existing commercial hazelnut/cocoa products and similar rheological behavior and sensory perception.

Even though specific environmental indicators were not calculated, the ecosustainability was addressed selecting locally available ingredients (carob and hazelnuts) rather than to imported ones (cocoa/cocoa powder and palm oil), and skins and grapeseed oil coming from the valorisation of grape-processing residues. The recipe formulation was at the same time driven by the need of improving the nutritional target in terms of fiber, unsaturated fat content, and antioxidant compounds. In fact, the developed spread could show the European “high unsaturated fat” “high fiber” nutritional claims, together with a higher total phenolic content and antioxidant capacity than commercial products.

In relation to the rheological behavior, at low temperatures, carob/hazelnut-based cream showed a lower viscosity than commercial spreads. However, this effect was minimized at higher temperatures, due to the occurrence of fat melting phenomena. Tribological results confirmed the higher fluidity of the new developed carob-based spread by lower friction values in the Stribeck curve. The use of grapeseed oil within the formulation determined a more fluid texture and higher lubricant and nutritional properties, despite imparting a higher thermal/oxidation susceptibility, as also testified by the lowest values of induction temperature and induction period than those observed for commercial spreads. In addition, the new spread exhibited a higher value in conjugated dienes, trienes, and p-anisidine values, due to the different nature of vegetable fat. To this end, the greater amount of unsaturated fats on total lipid fraction makes the product more sensible to the oxidation process, despite the higher total phenolic content and antioxidant capacity coming from carob and grape skin powders.

The proposed recipe could be replicated in different countries considering the increasing worldwide popularity of carob, while grape skins and grapeseed oil could be easily substituted by other locally available by-products, even though further investigation is needed to optimize formulation and structural parameters to better resemble commercial spread texture and improve stability over time. In relation to sensory profile, further steps can involve either recipe correction to mask/reduce the emerged bitter/sour characteristic attribute or consumers' tests to assess liking and acceptability connected with individual and social items to identify the best target consumer for this new spread.

## Figures and Tables

**Table 1:** Characterization of experimental (*S*_*A*_) and commercial spreads (*S*_*B*_ and *S*_*C*_) of Table S1 for total phenolic content (TPC as gallic acid equivalents (GAE)), specific (related to TPC) (TEAC_ABTS_) and total (related to spread mass) (TEAC′_ABTS_) antioxidant capacity by ABTS assay, and specific (FRAP) and total (FRAP′) antioxidant capacity by FRAP assay.

Sample	TPC (mg_GAE_/g_sample_)	TEAC_ABTS_ (mol_Trolox_/mol_GAE_)	TEAC′_ABTS_ (*μ*mol_Trolox_/g_sample_)	FRAP (mol_Fe(II)_/mol_GAE_)	FRAP′ (*μ*mol_Fe(II)_/g_sample_)
*S*_*A*_	9.49 ± 0.97^b^	1.60 ± 0.16^a^	87.74 ± 5.03^b^	5.18 ± 0.18^a^	285.95 ± 10.20^b^
*S*_*B*_	2.30 ± 0.33^a^	1.72 ± 0.24^a^	22.79 ± 0.48^a^	6.05 ± 0.57^a^	81.14 ± 7.70^a^
*S*_*C*_	1.91 ± 0.21^a^	1.91 ± 0.18^a^	21.27 ± 0.62^a^	6.67 ± 0.57^a^	21.27 ± 0.62^a^

Values are reported as mean values ± s.d. (*n* = 3). For each parameter, different lowercase letters indicate significantly different means (*p* < 0.05).

**Table 2:** Conjugated dienes (K_232_) and trienes (K_270_), p-anisidine (K_350_), and peroxide (meq_O2_/kg_oil_) values of the lipid fraction of experimental (*S*_*A*_) and commercial spreads (*S*_*B*_ and *S*_*C*_) of Table S1.

Lipid fraction of spread	K_232_	K_270_	K_350_	meq_O2_/kg_oil_
*S*_*A*_	2.64 ± 0.06^b^	2.04 ± 0.05^b^	10.43 ± 3.09^a^	N.D.
*S*_*B*_	N.D.	N.D.	N.D.	N.D.
*S*_*C*_	1.27 ± 0.03^a^	0.29 ± 0.04^a^	7.58 ± 3.99^a^	N.D.

Values are reported as mean values ± s.d. (*n* = 3). For each parameter, different lowercase letters indicate significantly different means (*p* < 0.05). N.D: not detected.

**Table 3:** Values of the main parameters obtained by elaboration of the DSC thermograms of experimental (*S*_*A*_) and commercial spreads (*S*_*B*_ and *S*_*C*_) of Table S1. For sugar-melting values of fusion enthalpy (Δ*H*), onset temperature (*T*_on_), peak temperature (*T*_peak_), and offset temperature (*T*_off_) were obtained.

Spread	Sugar melting				Lipid oxidation
	Δ*H* (J/g)	*T*_onset_ (°C)	*T*_peak_ (°C)	*T*_offset_ (°C)	IT (°C)
*S*_*A*_	8.53 ± 0.47^a^	125.81 ± 0.42^a^	136.52 ± 0.13^a^	142.21 ± 0.20^a^	144.50 ± 0.71^a^
*S*_*B*_	52.8 ± 0.95^c^	145.52 ± 0.17^c^	157.61 ± 0.17^c^	161.63 ± 0.15^c^	166.00 ± 1.41^c^
*S*_*C*_	44.8 ± 1.37^b^	137.83 ± 0.24^b^	155.32 ± 0.08^b^	158.82 ± 0.15^b^	162.50 ± 0.71^b^

For lipid oxidation, induction temperature (IT) was derived. Values are reported as mean values ± s.d. (*n* = 3 = 3). For each parameter, different lowercase letters indicate significantly different means (*p* < 0.05 < 0.05).

**Table 4:** Values of flow index (*n*) and consistency index (*K*) of experimental (*S*_*A*_) and commercial spreads (*S*_*B*_ and *S*_*C*_) of Table S1, obtained from the flow curve data regression by the power law model, at different temperatures.

Parameter	*T* (°C)	*S*_*A*_	*S*_*B*_	*S*_*C*_
*K* (Pa·s^n^)	10	54.7 ± 3.0^dA^	676.0 ± 20.0^dC^	513.0 ± 48.0^dB^
	20	38.5 ± 1.9^cA^	266.0 ± 14.0^cC^	205.0 ± 5.0^cB^
	30	27.7 ± 0.3^bA^	164.0 ± 3.0^bC^	122.0 ± 6.0^bB^
	40	23.5 ± 0.1^aA^	45.0 ± 3.0^aB^	76.0 ± 3.0^abC^
	50	23.4 ± 0.5^aA^	42.0 ± 2.0^aB^	71.0 ± 3.0^abC^
	60	29.9 ± 0.2^bA^	41.0 ± 2.0^aB^	63.0 ± 5.0^aC^

*n* (-)	10	0.582 ± 0.028^eB^	0.112 ± 0.002^aA^	0.130 ± 0.033^aA^
	20	0.561 ± 0.016^eB^	0.196 ± 0.006^bA^	0.299 ± 0.011^cA^
	30	0.514 ± 0.001^dC^	0.253 ± 0.009^cA^	0.287 ± 0.009^cB^
	40	0.439 ± 0.003^cC^	0.303 ± 0.011^dB^	0.265 ± 0.008^cA^
	50	0.350 ± 0.002^bC^	0.235 ± 0.014^cB^	0.189 ± 0.010^bA^
	60	0.269 ± 0.002^aB^	0.189 ± 0.016^bA^	0.199 ± 0.019^bA^

*R* ^2^	10	0.965	0.999	0.999
	20	0.975	0.997	0.999
	30	0.953	0.990	0.997
	40	0.958	0.981	0.981
	50	0.969	0.987	0.985
	60	0.974	0.985	0.988

Values are reported as mean values ± s.d. For each parameter, values with different lowercase letters within the same column (effect of temperature) are significantly different (*p* < 0.05), while values with different uppercase letters within the same row (effect of spread) are significantly different (*p* < 0.05).

**Table 5:** Values of the activation energy (*E*_*a*_) and preexponential factor (*η*_∞_), obtained from the flow curve data regression by the Arrhenius model at a fixed shear rate (50 s^−1^) and variable temperature (*T* = 10–60°C), for experimental (*S*_*A*_) and commercial spreads (*S*_*B*_ and *S*_*C*_) of Table S1.

	*E*_*a*_ (kJ/mol)	*η*_∞_ · 10^6^ (Pa·s)	*R* ^2^
*S*_*A*_	30.20 ± 0.55^a^	26.71 ± 5.29^b^	0.979
*S*_*B*_	42.11 ± 0.21^c^	0.36 ± 0.04^a^	0.962
*S*_*C*_	31.46 ± 0.04^b^	23.72 ± 0.17^b^	0.972

Values are reported as mean values ± s.d. For each parameter, different lowercase letters indicate significantly different means (*p* < 0.05).

## Data Availability

The data (analytical results collected in Excel files) used to support the findings of this study are available from the corresponding author upon reasonable request.

## Supplementary Materials

Supplementary Materials Supplementary material is provided for a better understanding of some parts of the paper and provides further insight. Figure S1 is explaining the experimental plan followed in the conduction of the present research. Figure S2 reports the curves obtained in the Oxitest analyses of the lipid fractions extracted from the three investigated spreads. Table S1 reports the ingredient list and nutritional composition of the three investigated spreads. For the newly developed spread, these were experimental data, while for the two commercial spreads, data was taken from the labels on the products. Table S2 reports the full fatty acid profile of the developed carob-based spread.
