# Supplementary material for: A Potentially Ecosustainable Hazelnut/Carob-Based Spread
Source: Int J Food Sci. 2024 Mar 14;2024:4863035. doi: 10.1155/2024/4863035 (PMC10957253; doi:10.1155/2024/4863035)
Supplement: Supplementary Materials — Supplementary material is provided for a better understanding of some parts of the paper and provides further insight. Figure S1 is explaining the experimental plan followed in the conduction of the present research. Figure S2 reports the curves obtained in the Oxitest analyses of the lipid fractions extracted from the three investigated spreads. Table S1 reports the ingredient list and nutritional composition of the three investigated spreads. For the newly developed spread, these were experimental data, while for the two commercial spreads, data was taken from the labels on the products. Table S2 reports the full fatty acid profile of the developed carob-based spread. [file 4863035.f1.docx]

**Supplementary Materials**


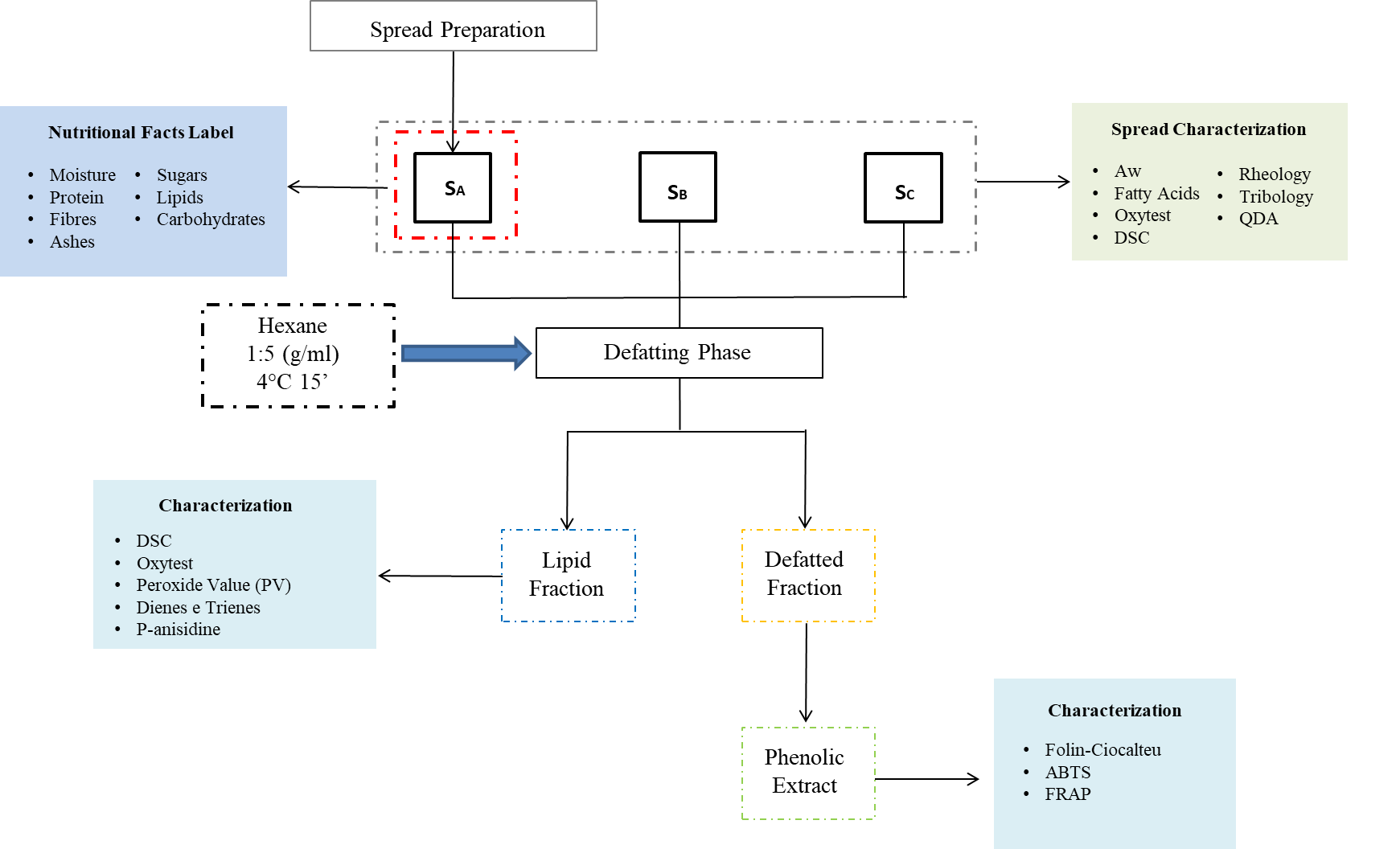


Figure S1: Schematic representation of the experimental protocol followed in this work, highlighting the characterization analysis and methodology applied to compare the newly produced spread (S_A_) with commercial ones (S_B_, and S_C_).

Figure S2: Curves of oxygen pressure (bar) reduction in the reactor chamber of the Oxitest system as a function of time for the lipid fractions of spreads of Table S1: S_A_ (solid line), S_B_ (dotted line), and S_C_ (dashed line). Tests were carried out at 100°C, with initial pure O_2_ pressure of 6 bar.

Table S1: Ingredients and nutritional information experimentally determined for the innovative spread S_A_ and taken from the label for the commercial spreads S_B_ and S_C_ tested in this work.

|  | S_A_^*^ | S_B_^*^ | S_C_^*^ |
| --- | --- | --- | --- |
|  | Nutritional information [per 100 g] | | |
| Energy [kcal – kJ] | 549 - 2278 | 539 - 2243 | 550 - 2300 |
| Proteins [g] | 11.2 | 6 | 10.4 |
| Carbohydrates [g] | 25.3 | 57.3 | 42.7 |
| *of which* sugars [g] | 24.9 | 56.8 | 40.7 |
| Fats [g] | 40.4 | 31.6 | 36 |
| *of which* saturated fats [g] | 0.1 | 10.9 | 5.8 |
| Fibres [g] | 19.8 | 3.4 | 6.9 |
| Sodium [mg] | 1 | 37 | 90 |

^*^Spreads composition: S_A_ (Carob flour (38.5%), GPO hazelnut paste (34%), grapeseed oil (17%), sugar (8%), grape-skin flour (2%), soy lecithin (0.5%), S_B_ (Sugar, vegetable oil (palm), hazelnut (13 %), low-fat cocoa powder (7.4 %), skimmed milk powder (6.6 %), emulsifier (soy lecithin), flavoring) and S_C_ (Hazelnuts (45 %), sugar, low-fat cocoa powder (9 %), skimmed milk powder (5 %), vegetable fat (cocoa butter), emulsifier (sunflower lecithin), flavoring)

Table S2. Fatty acid profile, expressed as the number of carbon atoms, for the experimental spread S_A_. SFAs: saturated fatty acids. UFAs: unsaturated fatty acids. MUFAc: monounsaturated fatty acids. PUFAs: polyunsaturated fatty acids.

| Fatty Acid | [g/100 g total fatty acid] | Fatty Acid | [g/100 g total fatty acid] |
| --- | --- | --- | --- |
| C 8:0 | 0.01 | C 18:2Δ^9,12^ | 37.91 |
| C 10:0 | 0.02 | C 18:3Δ^9,12,15^ | 0.26 |
| C 12:0 | 0.03 | C 20:0 | 0.18 |
| C 13:0 | 0.00 | C 20:1Δ^9^ | 0.14 |
| C 14:0 | 0.11 | C 22:0 | 0.10 |
| C 14:1Δ^9^ | 0.00 | C 22:1Δ^9^ | 0.00 |
| C 15:0 | 0.02 | C 24:0 | 0.05 |
| C 16:0 | 7.18 | SFAs | 11.36 |
| C 16:1Δ^9^ | 0.19 | UFAs | 88.64 |
| C 17:0 | 0.05 | of which MUFAs | 50.46 |
| C 18:0 | 3.61 | of which PUFAs | 38.18 |
| C 18:1Δ^9^ trans | 0.00 | ω-3 | 0.26 |
| C 18:1Δ^9^ | 50.13 | ω-6 | 37.91 |
